# The influence of prior practice and handedness on the orthogonal Simon effect

**DOI:** 10.3389/fpsyg.2014.00039

**Published:** 2014-02-07

**Authors:** Cristina Iani, Nadia Milanese, Sandro Rubichi

**Affiliations:** Department of Communication and Economics, University of Modena and Reggio EmiliaReggio Emilia, Italy

**Keywords:** orthogonal Simon effect, orthogonal spatial compatibility, handedness, practice paradigm, S-R associations

## Abstract

When stimuli are arranged vertically and responses horizontally, right-handed participants respond faster with right responses to stimuli presented above fixation and with left responses to stimuli presented below fixation, even when stimulus position is task-irrelevant (orthogonal Simon effect). The aim of the present work was twofold. First, we assessed whether the orthogonal Simon effect evident in right-handed participants is present also for left-handed participants (Experiment 1). Second, we investigated whether for both groups of participants the orthogonal Simon effect is influenced by the stimulus-response (S-R) mapping used for an orthogonal spatial S-R compatibility task performed 5 min before (Experiment 2). Our results showed that the orthogonal Simon effect significantly differed in the two groups, with left-handers showing an advantage for the up-left/down-right mapping (Experiment 1). Interestingly, the orthogonal Simon effect was strongly influenced by prior practice regardless of the participants’ handedness (Experiment 2). These results suggest that the short-term S-R associations acquired during practice can override the long-term, hardwired associations established on the basis of handedness.

## INTRODUCTION

It has been widely demonstrated that some stimulus-response (S-R) associations are easier to establish and faster to process than others. For instance, it has been shown that human performance is more efficient when stimuli and responses are ipsilateral, that is, on the same side (corresponding situations), than when they are contralateral (non-corresponding situations; e.g., Fitts and Seeger, 1953). The advantage for corresponding responses is evident even when stimulus location is irrelevant for performing the task and the response has to be emitted on the basis of a non-spatial stimulus feature (e.g., color or shape). For instance, when a right key has to be pressed in response to a red square and a left key has to be pressed in response to a green square, responses are faster if stimulus and response locations are on the same side as compared to when they are on different sides. The influence of the irrelevant spatial stimulus dimension on performance is known as Simon effect (Simon and Rudell, 1967; for a review see Proctor and Vu, 2006; Rubichi et al., 2006).

The Simon effect is considered an attentional phenomenon (e.g., Figliozzi et al., 2010) due to the interaction between two parallel and distinct processing routes, a slow conditional route and a fast unconditional route (e.g., Kornblum et al., 1990; De Jong et al., 1994), which are supposed to rely on different memory associations connecting stimuli and responses (Barber and O’Leary, 1997). When a stimulus appears, the slow conditional route activates the required response on the basis of task-defined associations connecting a stimulus to a specific response, while the fast unconditional route activates the response that spatially corresponds to the stimulus location through pre-existing stimulus–response associations, which are independent from instructions. When the two activated responses correspond, no competition arises. In contrast, when they are different, interference arises and the incorrect response needs to be aborted, affecting reaction times (RTs) and accuracy.

In the majority of the studies assessing spatial compatibility effects, both stimulus and response sets vary along the same dimension (see for example Rubichi et al., 2004, for a comparison between horizontal and vertical dimensions). However, these effects emerge even when “up” and “down” stimuli are mapped to left and right responses. This occurs both when stimulus location is relevant for task performance, a phenomenon known as orthogonal spatial compatibility (e.g., Weeks and Proctor, 1990; Lippa and Adam, 2001; Cho and Proctor, 2003; Proctor and Cho, 2006), and when it is irrelevant, a phenomenon known as orthogonal Simon effect (e.g., Cho and Proctor, 2005; Nishimura and Yokosawa, 2006; Cho et al., 2008). In both cases, even though there is not an evident spatial correspondence between stimuli and responses, performance is better when up stimuli are mapped to right responses and down stimuli are mapped to left responses (up-right/down-left mapping) than when up stimuli are mapped to left responses and down stimuli are mapped to right responses (up-left/down-right mapping).

To explain orthogonal correspondence effects, some authors proposed the so-called “asymmetric coding” account (e.g., Weeks and Proctor, 1990; Cho and Proctor, 2001, 2005) according to which stimulus and response alternatives with binary values are coded asymetrically as having a positive or a negative polarity. Specifically, “up” and “right” are coded as the polar referents for their relative dimensions (that is, up for the vertical dimension and right for the horizontal dimension) and hence coded with a positive polarity, while “down” and “left” are coded relative to up and right and hence with a negative polarity (see also Clark and Chase, 1972; Olson and Laxar, 1973; Seymour, 1974). Hence, performance is better for the up–right/down–left mapping because there is correspondence between polarity codes of stimulus and response dimensions (that is, correspondence of positive polarity for the up–right mapping and of negative polarity for the down-left one). Polarity coding occurs for any dimension that has two extreme poles and the term polarity is an arbitrary label used to refer to the binary way in which the two extreme poles are coded.

The finding of an orthogonal Simon effect (Nishimura and Yokosawa, 2006; Cho et al., 2008) implies that the long-term associations between stimulus and response codes of the same polarity lead to automatic response activation (Bae et al., 2009). Interestingly, these S-R links do not seem to be as strong as those between a stimulus and the spatially corresponding response. Indeed, when the parallel Simon task is performed after practicing a spatial compatibility task with an incompatible mapping between stimulus and response, the Simon effect disappears or even reverses (e.g., Proctor and Lu, 1999; Tagliabue et al., 2000; Rubichi et al., 2005; Iani et al., 2009; Lugli et al., 2013). This is thought to occur because the short-term associations between a stimulus location and the incompatible response that were created in order to perform the spatial compatibility task remain active and influence performance in the subsequent Simon task, hence contrasting the overlearned long-term associations (see Pellicano et al., 2010 for the effects of overlearned long-term associations). Interestingly, this “transfer of learning” effect occurs even when the practice task is observed and not performed (Iani et al., 2013). In contrast, practicing with a spatially compatible mapping does not affect the Simon effect hence suggesting that long-term links cannot be further improved by training.

By using a similar paradigm, Bae et al. (2009, Experiment 1) showed that prior practice with an orthogonal spatial compatibility task influences the orthogonal Simon effect with the two mappings exerting equal influences: a positive orthogonal Simon effect (that is, an advantage for the up-right/down-left relation) was found when participants practiced with the up-right/down-left mapping, while a reversed effect (that is, an advantage for the up-left/down-right relation) was found when participants practiced with the up-left/down-right mapping. The authors interpreted this result as an indication that the long-term links between codes of corresponding polarity are weaker than the long-term links between corresponding S-R locations influencing performance in the parallel Simon task.

A way to investigate the strength of these links is to assess whether they vary as a function of steady human features such as handedness. Manual laterality is considered as a defining characteristic of our species. It is indeed estimated that about 90% of the population prefers the right arm to the left when reaching for a target or manipulating objects (e.g., Peters, 1998; see Goble and Brown, 2008 for a review). Since manual laterality affects the way we interact with the world, it has been proposed that it also affects the way we represent information (e.g., Casasanto, 2009, 2011). Specifically, it has been shown that handedness influences external space representation (e.g., Sampaio and Chokron, 1992). Furthermore, it has been shown that individuals implicitly associate concepts with positive emotional valence with the side of body they could use more fluently (Casasanto, 2009). As regards, spatial compatibility effects, there is experimental evidence that the parallel Simon effect is influenced by handedness, with an advantage of the dominant hand when it executes a response in the corresponding space (e.g., Rubichi and Nicoletti, 2006; see also Rabbit, 1978; Peters, 1981). Ladavas (1987) demonstrated that also the orthogonal spatial S-R compatibility effect is affected by participants’ handedness. In her study, right-handed and left-handed participants performed an orthogonal spatial compatibility task in which upper and lower stimuli were mapped with left and right responses (the right hand pressed a right button and the left hand pressed a left button). Results showed better performance when both right-handers and left-handers participants responded to upper stimuli with the dominant hand and to lower stimuli with the non-dominant hand. That is, right-handed participants showed an advantage for the up-right/down-left mapping, whereas left-handers participants showed an advantage for the up-left/down-right mapping. The author explained these findings by postulating the existence of an asymmetry in the coding of the dominant and non-dominant hands along the vertical dimension with the dominant hand being represented as “up” and the non-dominant hand as “down”, irrespective of their actual position.

Based on the evidence described above, the present study was aimed at assessing whether handedness affects the orthogonal Simon effect and whether in left-handers the orthogonal Simon effect can be modulated by the type of mapping experienced in a prior orthogonal spatial compatibility task in which stimulus position was relevant. To this end, in Experiment 1, we assessed the orthogonal Simon effect in a group of right-handed participants and in a group of left-handed participants. In Experiment 2, we assessed whether the orthogonal Simon effect is influenced by the S-R mapping used for an orthogonal spatial S-R compatibility task performed 5 min before, irrespective of handedness.

## EXPERIMENT 1

As stated in the Introduction, the results by Ladavas (1987) indicate that, when stimulus position is task relevant, left-handers show a preferential association of the dominant hand with upper visual stimuli and of the non-dominant hand to lower visual stimuli. The aim of Experiment 1 was to assess whether, similarly to right-handers, left-handers show a preferential association of the dominant hand with upper visual stimuli and of the non-dominant hand to lower visual stimuli even when the stimulus position is task irrelevant.

Based on the results by Ladavas (1987) and according to the asymmetric coding account, we expected left-handers to show better performance when upper stimuli demand a left response and lower stimuli demand a right response. The finding of a reversed orthogonal Simon effect for left-handers would support the idea that the associations of stimulus and response codes of corresponding polarity are “hard wired”.

### METHOD

#### Participants

Forty undergraduate students of the University of Modena and Reggio Emilia took part in the experiment for either payment (7€) or course credit. Hand preference was assessed according to the Edinburgh Handedness Inventory (Oldfield, 1971) that produces scores ranging from +100 and –100. Twenty students were classified as right-handed (mean manual preference: 77.6, SD = 20.53; age range:19–30 years; 12 female) and twenty as left-handed (mean manual preference: –56.3, SD = 36.33; age range: 21–40 years; 12 female). Participants were naïve about the purpose of the study and reported having normal or corrected-to-normal visual acuity. The experiment was conducted in accordance with the guidelines laid down in the Declaration of Helsinki.

#### Apparatus and stimuli

Participants sat in front of a color monitor controlled by an IBM computer, in a dimly illuminated room, at a viewing distance of approximately 57 cm. The eyes were aligned to the center of the screen.

Stimulus presentation and response collection were controlled by E-prime software (Schneider et al., 2002). Stimuli were red or green squares (1.3° × 1.3°), which were randomly, presented 4° above or below a central white fixation cross (0.5° × 0.5°) on a dark background. Responses were executed by pressing the “D” and “L” keys on a standard keyboard with the left and right index fingers.

#### Procedure

Trials began with presentation of the fixation cross, accompanied by a 800 Hz warning tone. After 1 s, the imperative stimulus appeared above or below the fixation cross and remained visiblefor 1500 ms. The response terminated the trial. A 400-Hz tone was given for 500 ms following either an incorrect response or a late response (longer than 1500 ms). The next trial began 500 ms after the response or the feedback.

Participants were required to respond as quickly and accurately as possible to the color of the stimulus. Half of the right-handed and half of the left-handed participants responded to the red square with the right key and to the green square with the left key, the remaining participants experienced the opposite S-R mapping.

The task consisted of 360 trials divided in three blocks of 120 trials each, preceded by 24 practice trials.

### RESULTS

Responses that were 2 standard deviations above or below the participant’s mean were excluded from the analyses (3.5% of total trials).

Mean correct RTs and percentage of error (PE) were calculated for each participant as a function of correspondence (up-right and down-left mappings as corresponding; up-left and down-right mappings as non-corresponding) and submitted to two separate repeated-measures analyses of variance (ANOVA) with correspondence as within-subject factor and handedness (right-handers vs. left-handers) as between-subjects factor. Paired sample *t*-tests were employed as *post hoc* tests and the Bonferroni correction was applied so that the *p*-level was decreased to 0.025 for the first order interactions. The respective data are shown in **Table 1**.

**Table 1:** Experiment 1.

	Up-right/down-left mapping	Up-left/down-right mapping	Orthogonal Simon effect
Right-handers	405 (94.9)	407 (95.7)	2
Left-handers	390 (83.9)	383 (81.5)	-7

*Mean reaction time (and standard deviation) in ms for the orthogonal Simon task as a function of handedness (right-handers and left-handers), and mapping (up-right/down-left and up-left/down-right). The orthogonal Simon effect is computed as the difference between RTs in the up-left/down-right mapping and RTs in the up-right/down-left mapping.*

As regards RTs, the main effects of handedness, *F*(1,38) = 1.81, *p* = 0.19, and correspondence, *F*(1,38) = 1.33, *p* = 0.25, did not reach statistical significance. The interaction between correspondence and handedness reached significance, *F*(1,38) = 4.55, *p *< 0.05. *Post hoc* comparisons showed that for left-handers non-corresponding responses (383 ms) were significantly faster than corresponding responses (390 ms; p < 0.025). The difference between corresponding (405 ms) and non-corresponding (407 ms) responses did not reach significance for right-handers.

Overall PE was 4.7%. No significant main effect or interaction reached significance.

Our results showed that the orthogonal Simon effect significantly differed in the two groups, with left-handers showing a 7-ms advantage for the up-left/down-right mapping compared to the up-right/down-left mapping (that is, a reversed orthogonal Simon effect). These data suggest that a steady human feature such as handedness influences the polarity attributed to stimulus and response codes.

The finding of a non-significant effect for right-handers is not surprising. Indeed, previous studies assessing the orthogonal Simon effect in right-handers found an effect ranging from 3 to 12 ms (e.g., Nishimura and Yokosawa, 2006; Cho et al., 2008), thus suggesting that the long-term associations at the basis of this effect are not as strong as those responsible for the parallel Simon effect.

## EXPERIMENT 2

The main aim of the present experiment was to assess whether in left-handers the orthogonal Simon effect can be modulated by the type of mapping experienced in a prior orthogonal spatial compatibility task in which stimulus position was relevant. To this aim, right- and left-handers practiced an orthogonal spatial compatibility task with either an up-right/down-left or an up-left/down-right mapping. After a 5 min interval, they performed an orthogonal Simon task, in which stimulus color was relevant while stimulus position was irrelevant.

### METHOD

#### Participants

The same participants of Experiment 1 took part to Experiment 2. The time interval between the two experiments was 2 weeks. Participants for each handedness group were randomly assigned to the two different practice-mapping conditions: up-right/down-left mapping and up-left/down-right mapping. The experiment was conducted in accordance with the guidelines laid down in the Declaration of Helsinki.

#### Apparatus, stimuli and procedure

The apparatus was the same as in Experiment 1. Stimuli for the orthogonal spatial compatibility task were white squares (1.3° × 1.3°), randomly presented 4° above or below a central white fixation cross (0.5° × 0.5°) on a dark background. Responses were executed by pressing the “D” and “L” keys on a standard keyboard with the left and right index fingers. Stimuli and response keys for the orthogonal Simon task were the same used in Experiment 1.

Participants performed the orthogonal spatial compatibility task (from now on, practice session) followed, after a 5-min interval, by the same orthogonal Simon task (from now on, transfer session) performed in Experiment 1.

In the practice session, each trial began with the presentation of the fixation cross accompanied by a sound, followed after 500 ms by the imperative stimulus which was randomly shown above or below the fixation until a response was given, but anyway no longer than 1 s. A 400-Hz tone was given for 500 ms as feedback in case of errors: responses performed with the wrong key or slower than 1000 ms. The inter-trial interval was of 500 ms. Participants were required to respond as quickly and accurately as possible to stimulus position. 20 participants (10 right-handers and 10 left-handers) practiced with the up-right/down-left mapping; while the remaining 20 participants practiced with the up-left/down-right mapping. In the transfer session, participants performed the same orthogonal Simon task performed in Experiment 1, with the same mapping rule used in the first session.

The practice task consisted of 300 trials divided into three blocks of 100 trials each, preceded by 10 practice trials, while the transfer task consisted of 360 trials divided in three blocks of 120 trials each, preceded by 24 practice trials.

### RESULTS

Only the data for the Simon task were analyzed. For each participant, RTs shorter and longer than 2 standard deviations from the mean were excluded from the analyses (3.8% of the total trials).

Correct RTs and PE were submitted to two separate repeated-measures ANOVA with correspondence (up-right and down-left mappings as corresponding; up-left and down-right mappings as non-corresponding) as a within-subject factor, and practice mapping (up–right/down–left; up–left/down–right) and handedness as between-subjects factors. Paired sample *t*-tests were employed as *post hoc* tests and the Bonferroni correction was applied so that the *p*-level was decreased to 0.025 for the first order interactions. The respective data are displayed in **Table 2**.

**Table 2:** Experiment 2.

Practice mapping	Up-right/down-left	Up-left/down-right	Orthogonal Simon effect
**Right-handers**
Up-right/down-left	406 (98.0)	424 (93.9)	18
Up-left/down-right	422 (99.9)	404 (93.2)	-18
**Left-handers**
Up-right/down-left	372 (80.5)	383 (88.8)	11
Up-left/down-right	384 (79.8)	367 (71.7)	-17

*Mean reaction time (and standard deviation) in ms for the orthogonal Simon task as a function of practice mapping (up-right/down-left and up-left/down-right) and Simon mapping (up-right/down-left and up-left/down-right) for right- (top panel) and left-handers (bottom panel).The orthogonal Simon effect is computed as the difference between RTs in the up-left/down-right mapping and RTs in the up-right/down-left mapping.
*

Overall, left-handers were 20-ms faster than right-handers, as indicated by the main effect of handedness, *F*(1,36) = 9.17, *p *< 0.01. The main effects of correspondence, *F *< 1, and practice mapping, *F *< 1, did not reach statistical significance, however, they significantly interacted, *F*(1,36) = 54.56, *p*< 0.01. *Post hoc* tests showed that for the participants who practiced the up-right/down-left mapping, corresponding responses (389 ms) were significantly faster than non-corresponding responses (404 ms), this resulting in a 15-ms orthogonal Simon effect (*p*< 0.001). For the participants who practiced the up-left/down-right mapping, corresponding responses (403 ms) were significantly slower than non-corresponding responses (386 ms), this resulting in a 17-ms reversed orthogonal Simon effect (*p*< 0.001). Interestingly, this pattern of results was evident irrespective of handedness, as indicated by the lack of a significant correspondence × practice mapping × handedness interaction, *F* < 1. 

Overall PE was 4.3%. The analysis revealed only a significant correspondence × practice mapping interaction, *F*(1,36) = 21.20, *p *< 0.001. For the participants who practiced with the up-right/down-left mapping, responses were more accurate on up-right/down-left trials than on up-left/down-right trials (2.7% and 5.7% of errors, respectively, *p *< 0.01). For participants who practiced the up-left/down-right mapping, responses were more accurate on up-left/down-right trials than on up-right/down –left trials (2.5% and 6.2% of errors, respectively, *p *< 0.01). This two-way interaction was not modulated by handedness, *F *< 1.

Our results are consistent with the findings of Bae et al. (2009) in showing that the orthogonal Simon effect is influenced by the S-R associations between vertical stimulus positions and horizontal response locations established during practice on an orthogonal spatial compatibility task. Specifically, the orthogonal Simon effect was of 15 ms after practice with the up-right/down-left mapping and reversed to –17 ms after practice with the up-left/down-right mapping. Interestingly, practice with the up-right/down-left mapping increased the size of the orthogonal Simon effect in both right- and left-handers. Similarly, the up-left/down-right mapping reversed the effect in both right- and left-handers. Hence, it seems that the short-term S-R associations established during practice are strong enough to override the long-term associations responsible for the effect evident before performing the practice and that these long-terms associations are not unchangeable as those responsible for the parallel Simon effect.

### COMPARISON BETWEEN EXPERIMENTS

To further investigate the effect of the practice mapping, we submitted correct RTs of the two experiments to a repeated-measures ANOVA with practice mapping (up-right/down-left; up-left/down-right) and handedness as between-subject factors and correspondence (up-right and down-left mappings as corresponding; up-left and down-right mappings as non-corresponding) and session (Experiment 1 as session 1 and Experiment 2 as session 2) as within-subject factors. Paired sample *t*-tests were employed as *post hoc* tests and the Bonferroni correction was applied so that the *p*-level was decreased to 0.025 for the first order interactions.

This analysis revealed a significant main effect of handedness, *F*(1,36) = 5.09, *p *< 0.05, with faster RTs for left-handers (382 ms) than for right-handers (410 ms), and significant interactions between practice mapping and correspondence, *F*(1,36) = 26.62, *p *< 0.001, and between session, practice mapping, and correspondence, *F*(1,36) = 36.92, *p *< 0.001. Handedness did not interact with any factor. No other main effect or interaction reached statistical significance.

To further assess the three-way interaction, we performed separate analyses by practice mapping. These analyses showed that for the up-right/down-left practice mapping, the main effect of correspondence, *F*(1,18) = 13.38, *p *< 0.01, and the interaction between session and correspondence, *F*(1,18) = 21.97, *p *< 0.001, were significant. *Post hoc* comparisons indicated that the difference between corresponding and non-corresponding trials was significant only in Session 2, with corresponding trials (389 ms) being faster than non-corresponding trials (403 ms; *p *< 0.001). The main effect of correspondence, *F*(1,18) = 14.05, *p *< 0.01, and the interaction between session and correspondence, *F*(1,18) = 17.73, *p *< 0.01, were significant also for the up-left/down-right mapping. *Post hoc* comparisons indicated that the difference between corresponding and non-corresponding trials was significant only in Session 2, with non-corresponding trials (386 ms) being faster than corresponding trials (403 ms) (*p *< 0.001).

These results confirmed that practice with the up-right/down-left mapping increased the size of the orthogonal Simon effect in both right- and left-handers. Similarly, the up-left/down-right mapping reversed the effect in both right- and left-handers.

## GENERAL DISCUSSION

The aim of the present study was to assess whether the orthogonal Simon effect evident in right- and left-handers is affected in a similar way by the S-R mapping used in a prior orthogonal spatial compatibility task. Experiment 1 was designed to assess the orthogonal Simon effect in right-handed and left-handed participants, while Experiment 2 was aimed at assessing whether, for both sub-groups, the effect is influenced by the S-R mapping used for an orthogonal spatial S-R compatibility task performed 5 min before.

Our results showed that the orthogonal Simon effect significantly differed in left-handed compared to right-handed participants. While right-handers showed no reliable effect, left-handers showed an advantage for the up-left/down-right mapping (Experiment 1). This result supports the existence of asymmetries in spatial coding in both the vertical and horizontal dimension, which can be represented as polarity differences. As stated in the Introduction, stimulus and response alternatives with binary values are coded as having a positive or a negative polarity (e.g., Proctor and Cho, 2006). As regards the vertical dimension, there is indication that above tends to be coded as positive, and below as negative in vertical spatial representation, as also demonstrated by the finding that above positions are processed faster than below positions (e.g., Chase and Clark, 1971). As regards the horizontal dimension, right-handers code right as positive and left as negative, as suggested by faster processing of right positions as compared to left positions (e.g., Olson and Laxar, 1973, 1974). The findings of the present study, along with those of Nishimura and Yokosawa (2006) are consistent with the idea that handedness influences how we interact with the world and, as a consequence, the way we code and represent information (Casasanto, 2009) since they showed that the horizontal spatial representation is strongly affected by handedness. More precisely, the observation that, in the absence of prior practice, an advantage of the up-left/down-right mapping emerges for left-handers suggests that, differently from right-handers, they code left as positive and right as negative. While in right-handers the stimulus code of the above position automatically activates the right-response code that is the response code with the same polarity, in left-handers it automatically activates the left response code. Similarly, the stimulus code of the below position automatically activates the left response code in right-handers and the right response code in left-handers.

Interestingly, we showed that this tendency might be affected by prior practice. Indeed, the orthogonal Simon effect was strongly influenced by prior practice regardless of the participants’ handedness (Experiment 2). These results suggest that the long-term associations between stimulus and response codes of the same polarity established on the basis of handedness are weaker than the long-term spatially corresponding associations between stimulus and responses, which are thought to be overlearned or even genetically determined (e.g., Pellicano et al., 2010). Indeed, differently from the latter, that are unaffected by practice, long-term polarity associations can be easily overridden by the short-term S-R associations acquired during practice. To note, a recent study by Stock et al. (2013) showed that spatial aspects of a task can change patterns of information processing with spatial information being flexibly allocated to the two hemispheres of the brain. The results of the present study further extend this finding by showing that the flexible spatial representations formed during the practice session may affect how a subsequent (transfer) task is performed.

The finding that after practice left-handers displayed the same behavior as right-handers may be explained by invoking the asymmetries in lateralization observed between right- and left-handers. The results of several studies indicate that in hand motor skills right-handers show a stronger lateralization than left-handers, with right-handers relying more on their dominant hand as compared to left-handers who seem to equally rely on both hands (e.g., Kilshaw and Annett, 1983; Geschwind and Galaburda, 1987; Gonzalez et al., 2007; Linkenauger et al., 2009). In line with these findings, it has been shown that in right-handers the cortical representations of the right arm and hand are larger in the left-hemisphere than in the right hemisphere, while in left-handers there is a symmetrical representation across hemispheres (e.g., Linkenauger et al., 2009). Furthermore, there is recent evidence that cerebral laterality for spatial cognition differs between these two subgroups (Shimoda et al., 2008). Taken together, these differences in lateralization may explain why prior practice with a spatial compatibility task neutralized the differences in performance between right- and left-handers.

To conclude, the present data indicate that handedness might affect the way we code spatial information favoring specific associations between stimuli and responses that affect automatic response activation. However, short-term S-R associations acquired during a prior practice can easily override these associations established on the basis of a steady human feature such as handedness.

## Conflict of Interest Statement

The authors declare that the research was conducted in the absence of any commercial or financial relationships that could be construed as a potential conflict of interest.
